# Vowel Imagery Decoding toward Silent Speech BCI Using Extreme Learning Machine with Electroencephalogram

**DOI:** 10.1155/2016/2618265

**Published:** 2016-12-19

**Authors:** Beomjun Min, Jongin Kim, Hyeong-jun Park, Boreom Lee

**Affiliations:** Department of Biomedical Science and Engineering (BMSE), Institute of Integrated Technology (IIT), Gwangju Institute of Science and Technology (GIST), Gwangju, Republic of Korea

## Abstract

The purpose of this study is to classify EEG data on imagined speech in a single trial. We recorded EEG data while five subjects imagined different vowels, /a/, /e/, /i/, /o/, and /u/. We divided each single trial dataset into thirty segments and extracted features (mean, variance, standard deviation, and skewness) from all segments. To reduce the dimension of the feature vector, we applied a feature selection algorithm based on the sparse regression model. These features were classified using a support vector machine with a radial basis function kernel, an extreme learning machine, and two variants of an extreme learning machine with different kernels. Because each single trial consisted of thirty segments, our algorithm decided the label of the single trial by selecting the most frequent output among the outputs of the thirty segments. As a result, we observed that the extreme learning machine and its variants achieved better classification rates than the support vector machine with a radial basis function kernel and linear discrimination analysis. Thus, our results suggested that EEG responses to imagined speech could be successfully classified in a single trial using an extreme learning machine with a radial basis function and linear kernel. This study with classification of imagined speech might contribute to the development of silent speech BCI systems.

## 1. Introduction

People communicate with each other by exchanging verbal and visual expressions. However, paralyzed patients with various neurological diseases such as amyotrophic lateral sclerosis and cerebral ischemia have difficulties in daily communications because they cannot control their body voluntarily. In this context, brain-computer interface (BCI) has been studied as a tool of communication for these types of patients. BCI is a computer-aided control technology based on brain activity data such as EEG, which is appropriate for BCI systems because of its noninvasive nature and convenience of recording [1, 2].

The classification of EEG signals recorded during the motor imagery paradigm has been widely studied as a BCI controller [3–5]. According to these studies, different imagined tasks induce different EEG patterns on the contralateral hemisphere mainly in mu (7.5–12.5 Hz) and beta (13–30 Hz) frequency bands. Many researchers have successfully constructed BCI systems based on the limb movement imagination paradigm such as right hand, left hand, and foot movement [5–7]. However, EEG signals recorded during imagination of speech without any movement of either mouth or tongue are still difficult to classify; however, this topic has become an interesting issue for researchers because speech imagination has high similarity to real voice communication. For example, Deng et al. proposed a method to classify imagined syllables, /ba/ and /ku/, in three different rhythms using Hilbert spectrum methods, and the classification results were significantly greater than the chance level [8]. In addition, DaSalla et al. classified /a/ and /u/ as vowel speech imagery for EEG-based BCI [9]. Furthermore, a study to discriminate syllables embedded in spoken and imagined words using an electrocorticogram (ECoG) was conducted [10].

Obviously, for the BCI system, the use of optimized classification algorithms that categorize a set of data into different classes is essential, and these algorithms are usually divided into five groups: linear classifiers, neural networks, nonlinear Bayesian classifiers, nearest neighbor classifiers, and combinations of classifiers [11]. For instance, various algorithms for speech classification have been used, such as k-nearest neighbor classifier (KNN) [12], support vector machine (SVM) [9, 13], and linear discriminant analysis (LDA) [8].

The extreme learning machine (ELM) is a type of feedforward neural network for classification, proposed by Huang et al. [14]. ELM has high speed and good generalization performance compared to the classic gradient-based learning algorithms. There is growing interest in the application of ELM and its variants in the biomedical field, such as epileptic EEG pattern recognition [15, 16], MRI study [17], and BCI [18].

In this study, we measured the EEG activities of speech imagination and attempted to classify those signals using the ELM algorithm and its variants with kernels. In addition, we compared the results to the support vector machine with a radial basis function (SVM-R) kernel and linear discriminant analysis (LDA). As far as we know, applications of ELM as a classifier for EEG data of imagined speech have been rarely studied. In the present study, we will examine the validity of using ELM and its variants in the classification of imagined speech and the possibility of our method for applications in BCI systems based on silent speech.

## 2. Materials and Methods

### 2.1. Participants

Five healthy human participants (5 males; mean age: 28.25 ± 2.71, range: 26–32) participated in this study. All participants were native Koreans with normal hearing and right-handedness. None of the participants had any known neurological disorders or other significant health problems. All participants gave written informed consent, and the experimental protocol was approved by the Institutional Review Board (IRB) of the Gwangju Institute of Science and Technology (GIST). The approval process of the IRB complies with the declaration of Helsinki.

### 2.2. Experimental Paradigm

**Figure 1:**
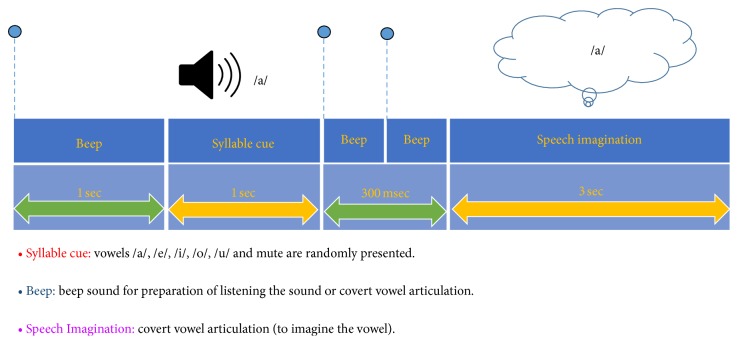
Schematic sequence of the experimental paradigm. Vowels /a/, /e/, /i/, /o/, /u/, and mute were randomly presented 1 s after the beginning of each trial. After the third beep sound, the subject imagines the same vowel heard at the beginning of the trial. The EEG data acquired during the speech imagination period were used for signal processing and classification in this study.

Participants were seated in a comfortable armchair and wore earphones (er-4p, Etymotic research, Inc., IL 60007, United States of America) providing auditory stimuli. Five types of Korean syllables—/a/, /e/, /i/, /o/, and /u/, as well as a mute (zero volume) sound—were utilized in the experiment.  Figure 1 describes the overall experimental paradigm. At the beginning of each trial, a beep sound was presented to prepare the participants for perception of the target syllable. These six auditory cues (including the mute sound) were recorded using Goldwave software (GoldWave, Inc., St. John's, Newfoundland, Canada), and the source audio was from Oddcast's online (http://www.oddcast.com/home/demos/tts/tts_example.php?sitepa). The five vowels and mute sound were randomly presented. Another 1 s after the onset of the target syllable, two beep sounds were given sequentially, with a 300 ms interval between them. After the two beep sounds, participants were instructed to imagine the same syllable heard at the beginning of the trial. The time for imagination was 3 s for each trial. Participants performed 5 sessions, with each session consisting of 10 trials for each syllable. Resting times were given between sessions for 1 min. Therefore, 50 trials were recorded for each syllable and the mute sound, and the total time for the experiment was approximately 10 min. All sessions were carried out in a day.

The experimental procedure was designed with e-Prime 2.0 software (Psychology Software Tools, Inc., Sharpsburg, PA, USA). A HydroCel Geodesic Sensor Net with 64 channels and Net Amps 300 amplifiers (Electrical Geodesics, Inc., Eugene, OR, USA) were used to record the EEG signals, using a 1000 Hz sampling rate (Net Station version 4.5.6).

### 2.3. Data Processing and Classification Procedure

#### 2.3.1. Preprocessing

First, we resampled the acquired EEG data into 250 Hz for fast preprocessing procedure. The EEG data was bandpass filtered with 1–100 Hz. Sequentially, an IIR notch filter (Butterworth; order: 4; bandwidth: 59–61 Hz) was applied to remove the power line noise.

**Figure 2:**
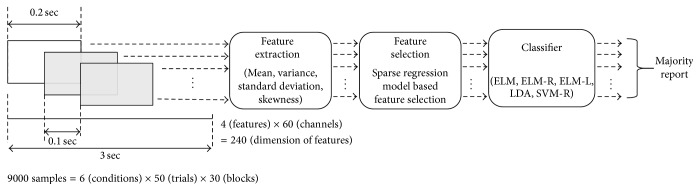
Overall signal processing procedure for classification. First, each trial was divided into thirty blocks with a 0.2 s length and 0.1 s overlap. Mean, variance, standard deviation, and skewness were extracted from all blocks and channels. Sequentially, sparse-regression-model-based feature selection was employed to reduce the dimension of the features. All features were used as the input of the trained classifier. Because each trial includes thirty blocks, thirty classifier outputs were acquired; therefore, the label of each trial was determined by selecting the most frequent output of the thirty classifier outputs.

In general, EEG classification has problems in terms of poor generalization performance and the overfitting phenomenon because the number of samples is much smaller than the dimension of the features. Therefore, to obtain enough samples for learning and testing the classifier, we divided each imagination trial for 3 s into 30 time segments with a 0.2 s length and 0.1 s overlap. Therefore, we obtained a total of 9000 segments = (6 (conditions) × 50 (trials per each condition) × 30 segments) to learn and test the classifier. We calculated the mean, variance, standard deviation, and skewness from each segment to acquire the feature vector for the classifier. The dimension of the feature vector is 240 (4 (types of features) × 60 (the number of channels)). Additionally, to reduce the dimension of the feature vector, we applied a feature selection algorithm based on the sparse regression model. The selected set of features extracted from all segments was employed to learn and test the classifier. Because a trial consists of thirty segments, a trial has thirty outputs of the classifier. Therefore, the label of the test trial was determined by selecting the most frequent output among the outputs of the thirty segments. The training and testing of the classifier model are conducted using the segments extracted only from training data and testing data, respectively. Finally, to accurately estimate the classification performance, we applied 10-fold cross-validation. The classification accuracies of ELM, extreme learning machine with linear function (ELM-L), extreme learning machine with radial basis function (ELM-R), and SVM-R for all five subjects were compared to select the optimal classifier to discriminate the vowel imagination. The overall signal processing procedures are briefly described in Figure 2.

#### 2.3.2. Sparse-Regression-Model-Based Feature Selection

Tibshirani developed a sparse regression model known as the Lasso estimate [19]. In this study, we employed the sparse regression model to select the discriminative set of features to classify the EEG responses to covert articulation. The formula for selecting discriminative features based on the sparse regression model can be described as follows:

(1) $$\begin{array}{c} z^{*}=\underset{z}{\mathrm{argmin}}{\left\|\;Fz-\overset{-}{t}\;\right\|}_{2}^{2}+\lambda {\left\|\;z\;\right\|}_{1}, \end{array}$$

where ‖·‖_*p*_ denotes the *l*_*p*_-norm, **z** is a sparse vector to be learned, and **z** ^**∗**^ indicates an optimal sparse vector. $\overset{-}{t}\in R^{N_{t}\times 1}$ is a vector about the true class label for the number of training samples, *N*_*t*_, and *λ* is a positive regularization parameter that controls the sparsity of **z**. **F** is the matrix that consists of the mean, variance, standard deviation, and skewness for each channel

(2) $$\begin{array}{c} F=\left[\;f_{1},f_{2},\ldots ,f_{240}\;\right], \end{array}$$

where **f**_*p*_ ∈ *ℜ* ^*N*_*t*_×1^ is the *p*th column vector of **F**. The coordinate descent algorithm is adopted to solve the optimization problem in (1) [20].

The column vectors in **F** corresponding to the zero entries in **z** are excluded to form an optimized feature set, $\tilde{F}$, that is of lower dimensionality than **F**.

#### 2.3.3. Extreme Learning Machine

Conventional feedforward neural networks require weights and biases for all layers to be adjusted by the gradient-based learning algorithms. However, the procedure for tuning the parameters of all layers is very slow because it is repeated many times, and its solutions easily fall into local optima. For this reason, Huang et al. proposed ELM, which randomly assigns the input weights and analytically calculates only the output weights. Therefore, the learning speed of ELM is much faster than conventional learning algorithms and has outstanding generalization performance [21–23]. If we assume the *N*_*t*_ training samples {(**v**_*k*_, **l**_*k*_)}_*k*=1_^*N*_*t*_^, where **v**_*k*_ is an *n*-dimensional feature vector, **v**_*k*_ = [*v*_*k*,1_, *v*_*k*,2_,…,*v*_*k*,*n*_]^*T*^, and **l**_*k*_ is the true labels, which consists of *m*-classes, **l**_*k*_ = [*l*_*k*1_, *l*_*k*2_,…,*l*_*km*_]^*T*^, a standard SLFN with *N*_*h*_ hidden neurons and activation function *a*(·) can be formulated as follows:

(3) $$\begin{array}{c} \overset{N_{h}}{\underset{j=1}{\sum }}w_{j}^{h}a\left(\;w_{j}^{i}\cdot v_{k}+b_{j}\;\right)=o_{k},\;k=1,\ldots ,N_{t}, \end{array}$$

where **w**_*j*_^*i*^ = [*w*_*j*,1_^*i*^, *w*_*j*,2_^*i*^,…,*w*_*j*,*n*_^*i*^]^*T*^ is the weight vector for the input layer between the *j*th hidden neuron and the input neurons, **w**_*j*_^*h*^ = [*w*_*j*,1_^*h*^, *w*_*j*,2_^*h*^,…,*w*_*j*,*m*_^*h*^]^*T*^ is the weight vector for the hidden layer between the *j*th hidden neuron and the output neurons, **o**_*k*_ = [*o*_*k*,1_, *o*_*k*,2_,…,*o*_*k*,*m*_]^*T*^ is the output vector of the network, and **b**_*j*_ is the bias of the *j*th hidden neuron. The operator · indicates the inner product. We can now reformulate the equation into matrix form as follows

(4) $$\begin{array}{c} AW^{h}=O, \end{array}$$

where

(5) A=aw1i·v1+b1⋯awNhi·v1+bNh⋮⋯⋮aw1i·vNt+b1⋯awNhi·vNt+bNhNt×Nh,Wh=w1h⋯wNhhNh×mT,O=o1⋯oNtNt×mT,

where matrix **A** is the output matrix of the hidden layer and the operator ^*T*^ indicates the transpose of the matrix. Because the ELM algorithm randomly selects the input weights **w**_*j*_^*i*^ and biases **b**_*j*_, we can find weights for the hidden layer, **w**_*j*_^*h*^, by solving the following optimization problem:

(6) $$\begin{array}{c} \underset{w_{j}^{h}}{\min}\;{\left\|\;AW^{h}-L\;\right\|}^{2}, \end{array}$$

where **L** is the matrix of true labels for training samples

(7) $$\begin{array}{c} L={\left(\;\begin{array}{ccc} l_{1} & \cdots & l_{N_{t}} \end{array}\;\right)}_{N_{t}\times m}^{T}. \end{array}$$

The above problem is known as a linear system optimization problem, and its unique least-squares solution with a minimum norm is as follows:

(8) $$\begin{array}{c} \hat{W^{h}}=A^{†}L, \end{array}$$

where **A** ^†^ is the Moore–Penrose generalized inverse of the matrix **A**. According to the analysis of Bartlett and Huang, the ELM algorithms achieve not only the minimum square training error but also the best generalization performance on novel test samples [14, 24].

In this paper, the activation function *a*(·) was determined to be a sigmoidal function, and the probability density function for assigning the input weights and biases was set to be a uniform distribution function.

## 3. Results and Discussion

### 3.1. Time-Frequency Analysis for Imagined Speech EEG Data

**Figure 3:**
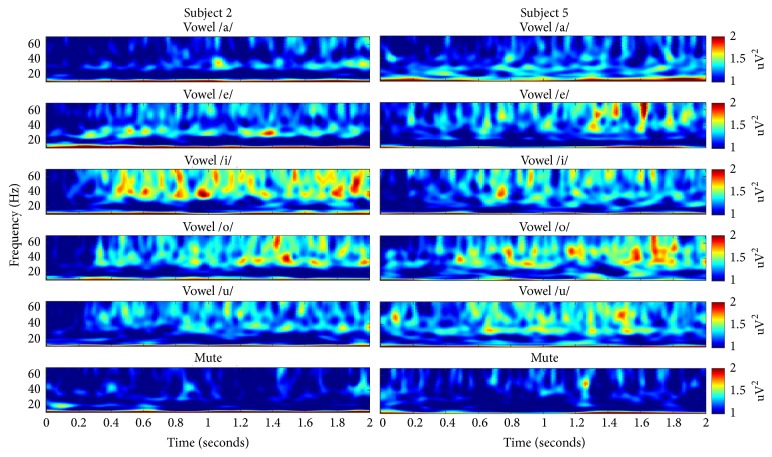
Time-frequency representation (TFR) of EEG signals averaged over all trials for subjects 2 and 5. The EEG signals were obtained from eight electrodes in the left temporal areas during each of the six experimental conditions (vowels /a/, /e/, /i/, /o/, /u/, and mute). The EEG data were bandpass filtered with 1–100 Hz, and a Morlet mother wavelet transform was used to calculate the TFR. The TFRs are plotted for the first 2 s after final beep sound and for the frequency range of 10–70 Hz.

**Figure 4:**
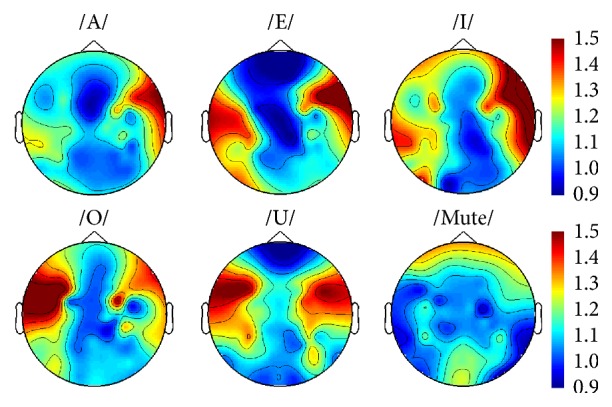
Topographical distribution of gamma activities during vowel imagination for subject 5. Increased activities were observed in both temporal areas when the subject imagined vowels. Time interval for the analysis is 0–3 sec.

We computed the time-frequency representation (TFR) of imagined speech EEG data for every subject to identify speech-related brain activities. TFR of each trial was calculated using a Morlet wavelet and averaged over all trials. Among the five subjects, we plotted TFRs of subjects 2 and 5 which showed notable patterns in gamma frequency. As shown in Figure 3, much of the gamma band (30–70 Hz) powers of five vowel conditions (/a/, /e/, /i/, /o/, and /u/) in the left temporal area are totally distinct and much higher than those of the control condition (mute sound). In addition, topographical head plot of subject 5 was presented in Figure 4. Increased gamma activities were observed in both temporal regions when the subject imagined vowels.

### 3.2. Classification Results

**Figure 5:**
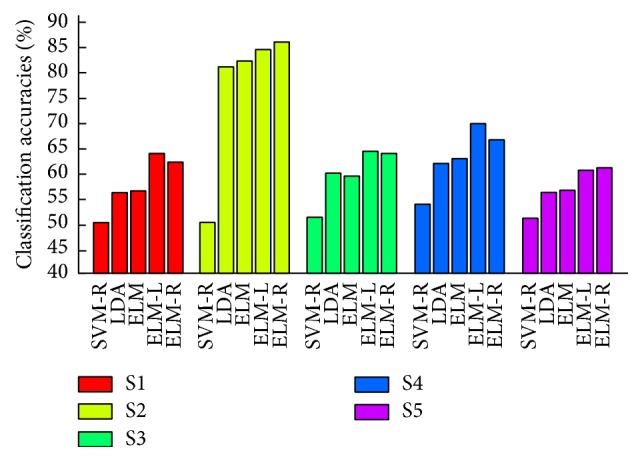
Averaged classification accuracies over all pairwise classification using a support vector machine with a radial basis function kernel (SVM-R), extreme learning machine (ELM), extreme learning machine with a linear kernel (ELM-L), and extreme learning machine with a radial basis function kernel (ELM-R) for all five subjects.

Figure 5 shows the classification accuracies averaged over all pairwise classifications for five subjects using ELM, ELM-L, ELM-R, SVM-R, and LDA. We also conducted SVM and SVM with a linear kernel, but the results of SVM and SVM with a linear kernel are excluded because these classifiers could not be converged during many iterations (100,000 times). All classification accuracies are estimated by 10 × 10-fold cross-validation. In the cases of subjects 1, 3, and 4, ELM-L shows the best classification performance compared to the other four classifiers. However, ELM-R shows the best classification accuracies in subjects 2 and 5. In the cases of all subjects, the classification accuracies of ELM, ELM-L, and ELM-R are much better than those of SVM-R, which are approximately the chance level of 50%. To identify the best classifier to discriminate the vowel imagination, we conducted paired *t*-tests between the classification accuracies of ELM-R and those of the other three classifiers. As a result, the classification performance of ELM-R is significantly better than those of ELM (*p* < 0.01), LDA (*p* < 0.01), and SVM-R (*p* < 0.01). However, there is no significant difference between the classification accuracies of ELM-R and ELM-L (*p* = 0.46).

Table 1 describes the classification accuracies of subject 2, which shows the highest overall accuracies among all subjects, after 10 × 10-fold cross-validation, for all pairwise combinations. In almost all pairwise combinations, ELM-R has better classification performance than the other four classifiers for subject 2. The most discriminative pairwise combination for subject 2 is vowels /a/ and /i/, which shows 100% classification accuracy using ELM-R for subject 2.

Table 2 contains the results of ELM-R for the pairwise combinations and shows the top five classification performances for each subject. There is no pairwise combination to be selected from all subjects; however, /a/ versus mute and /i/ versus mute are selected from four subjects, and /a/ versus /i/ is selected from three subjects.

Table 3 indicates the confusion matrix for all pairwise combinations and subjects using ELM, ELM-L, ELM-R, SVM-R, and LDA. In terms of sensitivity and specificity, ELM-L is the best classifier for our EEG data. Although SVM-R shows higher specificity than those of the other three classifiers in this table, SVM-R classified almost all conditions as positive and resulted in poor sensitivity; therefore, the high specificity of the SVM-R is possibly invalid. Thus, SVM-R might be an unsuitable classifier for our study.

### 3.3. Discussion

Overall, ELM, ELM-L, and ELM-R showed better performance than the SVM-R and LDA algorithms in this study. In several previous studies, ELM achieved similar or better classification accuracy rates with much less training time compared to other algorithms using EEG data [16, 25–27]. However, we could not find studies on classification of imagined speech using ELM algorithms. Deng et al. reported classification rates using LDA for imagined speech with 72.67% of the highest accuracy, but the average results were not much better than the chance level [8]. DaSalla et al. using SVM showed approximately 82% of the best accuracy and 73% of the average result overall [9], whereas Huang et al. reported that ELM tends to have a much higher learning speed and comparable generalization performance in binary classification [21]. In another study, Huang argued that ELM has fewer optimization constraints owing to its special separability feature and results in simpler implementation, faster learning, and better generalization performance [23]. Thus, our results showed consistent characters with others' previous research using ELM and even similar or better classification results for imagined speech compared to other research using different algorithms. Recently, ELM algorithms have been extensively applied in many other medical and biomedical studies [28–31]. More detailed information about ELM can be found in a recent review [32].

In this study, each trial was divided into the thirty time segments of 0.2 s in length and a 0.1 s overlap. Each time segment was considered as a sample for training the classifier, and the final label of the test sample was determined by selecting the most frequent output (see Figure 2). We also compared the classification accuracy of our method with those of a conventional method that does not divide the trials into multiple time segments. As a result, our method showed superior performance in terms of classification accuracy to the conventional method. In our opinion, by dividing the trials, some effects such as increasing number of trials for classifier training might occur, and each time segment with a 0.2 s length is likely to retain enough information for discrimination of EEG vowel imagination. Generally, EEG classification has problems in terms of poor generalization performance and the overfitting phenomenon because of the deficiency of the number of samples for the classifier. Therefore, an increased number of samples by dividing trials could mitigate the aforementioned problems. However, further analyses are required to prove our assumptions in subsequent studies.

**Figure 6:**
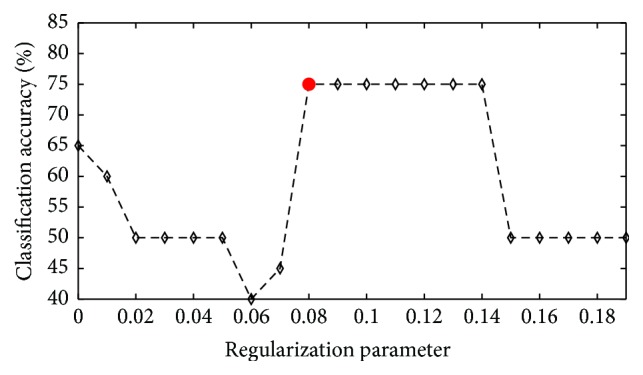
Effects of varying the regularization parameter on the classification accuracies obtained by ELM-R with sparse-regression-model-based feature selection for subject 2. The parameter value giving the highest accuracy is highlighted with a red circle.

To reduce the dimension of the feature vector, we employed a feature selection algorithm based on the sparse regression model. In the sparse-regression-model-based feature selection algorithm, the regularization parameter, *λ*, of equation (1) must be carefully selected because *λ* determines the dimension of the optimized feature parameter. For example, when the selected *λ* is too large, the algorithm excludes discriminative features from an optimal feature set, $\tilde{F}$. However, when users set *λ* too small, redundant features are not excluded from an optimal feature set $\tilde{F}$. Therefore, the optimal value for *λ* was selected by cross-validation on the training session in our study. For example, the change of classification accuracy caused by varying *λ* for subject 1 is illustrated in Figure 6. In the case of /a/ and /i/ using ELM-R, the best classification accuracy reached a plateau at *λ* = 0.08 and declined after 0.14. However, the optimal values of *λ* are totally different among the pairwise combinations and all subjects.

Furthermore, our optimized results were achieved in the gamma frequency band (30–70 Hz). We also tested the other frequency ranges, such as beta (13–30 Hz), alpha (8–13 Hz), and, theta (4–8 Hz); however, the classification rates of those bands were not much better than the chance level in every subject and pairwise combination of syllables. In addition, the results of our TFR and topographical analysis (Figures 3 and 4) could support some relationship between gamma activities and imagined speech processing. As far as we know, in the EEG classification of imagined speech, there have been only a few studies that examined the differences between multiple frequency bands including gamma frequency [33, 34]. Therefore, our study might be the first report that the gamma frequency band could play an important role as features for the EEG classification of imagined speech. Moreover, several studies using ECoG reported quite good results in the gamma frequency for imagined speech classification [35, 36], and these findings are consistent with our results. However, several studies have been conducted that suggested the role of gamma frequency band for speech processing in neurophysiological perspectives [37–39]. However, those studies usually used intracranial recordings and focused on the analysis for the high gamma (70–150 Hz) frequency band. Thus, suggesting a relevance between those results and our classification study is not easy. However, a certain relation between some information in low gamma frequencies as a feature for classification and its implication from a neurophysiological view will be specified in future studies.

Currently, communication systems with various BCI technologies have been developed for disabled people [40]. For instance, the P300 speller is one of the most widely researched BCI technologies to decode verbal thoughts from EEG [41]. Despite many efforts toward better and faster performance, the P300 speller is still insufficient for use in normal conversation [42, 43], whereas, independent of the P300 component, efforts toward extraction and analysis of EEG or ECoG induced by imagined speech have been conducted [44, 45]. In this context, our results of high performance from the application of ELM and its variants have potential to advance BCI research using silent speech communication. However, the pairwise combinations with the highest accuracies (see Table 2) differed in each subject. After experiment, each participant reported different patterns of vowel discrimination. For example, one subject reported that he could not discriminate /e/ from /i/, and the other subject reported the other pair was not easy to distinguish. Although those reports were not exactly matched to the results of classification, these discrepancies of subjective sensory perception might be related to process of imagining speech and classification results. Besides, we have not tried multiclass classification in this study, yet some attempts in multiclass classification of imagined speech have been performed by others [8, 46, 47]. These issues related to intersubject variability and multiclass systems should be considered for our future study to develop more practical and generalized BCI systems using silent speech.

## 4. Conclusions

In the present study, we used classification algorithms for EEG data of imagined speech. Particularly, we compared ELM and its variants to SVM-R and LDA algorithms and observed that ELM and its variants showed better performance than other algorithms with our data. These results might lead to the development of silent speech BCI systems.

## Figures and Tables

**Table 1:** Classification accuracies in % employing SVM-R, ELM, ELM-L, ELM-R, and LDA for subject 2. The highest classification accuracy among the four classifiers is marked in bold for pairwise combination. Classification accuracies are expressed as mean and associated standard deviation. SVM-R, ELM, ELM-L, ELM-R, and LDA denote the support vector machine with radial basis function, extreme learning machine, extreme learning machine with a linear kernel, extreme learning machine with a radial basis function, and linear discriminant analysis, respectively.

Classifier	/a/ versus /e/	/a/ versus /i/	/a/ versus /o/	/a/ versus /u/	/e/ versus /i/	/e/ versus /o/	/e/ versus /u/	/i/ versus /o/	/i/ versus /u/	/o/ versus /u/	/a/ versus /mute/	/e/ versus /mute/	/i/ versus /mute/	/o/ versus /mute/	/u/ versus /mute/
SVM-R	50.24 ± 1.01	55.32 ± 2.15	51.41 ± 0.51	50.22 ± 0.60	50.31 ± 0.19	49.18 ± 0.23	52.41 ± 1.57	52.47 ± 0.71	51.11 ± 0.22	51.02 ± 1.32	49.48 ± 0.70	50.35 ± 0.22	51.22 ± 1.22	50.14 ± 1.60	51.23 ± 0.23
ELM	81.41 ± 1.18	94.23 ± 1.02	62.42 ± 2.15	73.34 ± 3.21	90.55 ± 1.78	69.76 ± 12.94	56.23 ± 3.48	96.32 ± 0.18	97.47 ± 0.76	66.81 ± 3.83	92.85 ± 2.43	82.31 ± 2.16	99.41 ± 0.12	88.16 ± 3.74	80.28 ± 2.87
ELM-L	81.32 ± 0.47	98.15 ± 0.67	67.11 ± 1.04	82.22 ± 2.40	88.31 ± 1.73	78.25 ± 2.23	53.14 ± 3.10	98.16 ± 0.37	98.23 ± 2.12	68.36 ± 1.63	92.28 ± 0.83	80.49 ± 3.70	99.12 ± 0.33	93.14 ± 2.21	87.25 ± 1.12
ELM-R	86.28 ± 1.12	99.02 ± 0.76	73.03 ± 4.62	83.14 ± 1.02	89.08 ± 0.14	78.27 ± 0.16	58.36 ± 3.41	98.15 ± 0.22	97.07 ± 1.42	73.38 ± 2.73	95.09 ± 1.03	89.28 ± 3.13	99.01 ± 0.14	93.25 ± 0.73	93.01 ± 1.12
LDA	79.25 ± 1.62	90.32 ± 2.61	60.57 ± 2.13	84.12 ± 1.14	88.23 ± 1.67	70.04 ± 1.43	56.38 ± 1.41	97.07 ± 0.39	96.28 ± 1.62	65.14 ± 1.73	91.26 ± 1.43	80.38 ± 4.70	98.07 ± 1.32	90.39 ± 1.62	82.25 ± 1.27

**Table 2:** Classification accuracies in % employing ELM-R for the pairwise combinations, which shows the top five classification performances for each subject. Classification accuracies are expressed as mean and associated standard deviation.

Subjects					
S1	86.47 ± 1.07	81.21 ± 1.03	80.01 ± 3.73	73.35 ± 3.17	72.44 ± 1.71
	(/a/ versus /i/)	(/a/ versus mute)	(/a/ versus /u/)	(/a/ versus /e/)	(/i/ versus /o/)
S2	99.02 ± 0.76	99.30 ± 0.14	98.22 ± 0.22	95.14 ± 1.03	93.01 ± 0.73
	(/a/ versus /i/)	(/i/ versus mute)	(/i/ versus /o/)	(/a/ versus mute)	(/o/ versus mute)
S3	92.08 ± 1.08	90.19 ± 0.63	89.15 ± 1.37	87.27 ± 0.71	70.38 ± 1.38
	(/e/ versus mute)	(/i/ versus mute)	(/u/ versus mute)	(/o/ versus mute)	(/a/ versus /i/)
S4	93.33 ± 0.31	92.27 ± 1.03	92.24 ± 2.13	91.12 ± 0.54	90.05 ± 1.83
	(/i/ versus mute)	(/u/ versus mute)	(/a/ versus mute)	(/e/ versus mute)	(/o/ versus mute)
S5	96.32 ± 2.31	94.01 ± 0.17	92.29 ± 1.14	90.07 ± 0.58	88.06 ± 1.23
	(/e/ versus mute)	(/i/ versus mute)	(/o/ versus mute)	(/a/ versus mute)	(/u/ versus mute)

**Table 3:** Confusion matrix for all pairwise combinations and subjects using ELM, ELM-L, ELM-R, SVM-R, and LDA.

	Classifiers									
	ELM		ELM-L		ELM-R		SVM-R		LDA	
	Condition positive	Condition negative	Condition positive	Condition negative	Condition positive	Condition negative	Condition positive	Condition negative	Condition positive	Condition positive
Test positive	2516	1234	2649	1101	2635	1115	3675	75	2556	1194

Test negative	1509	2241	1261	2489	1297	2453	3525	225	1398	2352

	Sensitivity = 0.6251	Specificity = 0.6449	Sensitivity = 0.6775	Specificity = 0.6933	Sensitivity = 0.6701	Specificity = 0.6875	Sensitivity = 0.5104	Specificity = 0.7500	Sensitivity = 0.6464	Specificity = 0.6633

## References

[1] Hwang H.-J., Kim S., Choi S., Im C.-H. (2013). EEG-based brain-computer interfaces: a thorough literature survey. *International Journal of Human-Computer Interaction*.

[2] Chaudhary U., Birbaumer N., Ramos-Murguialday A. (2016). Brain–computer interfaces for communication and rehabilitation. *Nature Reviews Neurology*.

[3] Hamedi M., Salleh S.-H., Noor A. M. (2016). Electroencephalographic motor imagery brain connectivity analysis for BCI: a review. *Neural Computation*.

[4] Neuper C., Scherer R., Wriessnegger S., Pfurtscheller G. (2009). Motor imagery and action observation: modulation of sensorimotor brain rhythms during mental control of a brain-computer interface. *Clinical Neurophysiology*.

[5] Hwang H.-J., Kwon K., Im C.-H. (2009). Neurofeedback-based motor imagery training for brain–computer interface (BCI). *Journal of Neuroscience Methods*.

[6] Pfurtscheller G., Brunner C., Schlögl A., Lopes da Silva F. H. (2006). Mu rhythm (de)synchronization and EEG single-trial classification of different motor imagery tasks. *NeuroImage*.

[7] Ahn M., Jun S. C. (2015). Performance variation in motor imagery brain-computer interface: a brief review. *Journal of Neuroscience Methods*.

[8] Deng S., Srinivasan R., Lappas T., D'Zmura M. (2010). EEG classification of imagined syllable rhythm using Hilbert spectrum methods. *Journal of Neural Engineering*.

[9] DaSalla C. S., Kambara H., Sato M., Koike Y. (2009). Single-trial classification of vowel speech imagery using common spatial patterns. *Neural Networks*.

[10] Pei X., Barbour D. L., Leuthardt E. C., Schalk G. (2011). Decoding vowels and consonants in spoken and imagined words using electrocorticographic signals in humans. *Journal of Neural Engineering*.

[11] Lotte F., Congedo M., Lécuyer A., Lamarche F., Arnaldi B. (2007). A review of classification algorithms for EEG-based brain-computer interfaces. *Journal of Neural Engineering*.

[12] Brigham K., Kumar B. V. K. V. Imagined speech classification with EEG signals for silent communication: a preliminary investigation into synthetic telepathy.

[13] Kim J., Lee S.-K., Lee B. (2014). EEG classification in a single-trial basis for vowel speech perception using multivariate empirical mode decomposition. *Journal of Neural Engineering*.

[14] Huang G.-B., Zhu Q.-Y., Siew C.-K. (2006). Extreme learning machine: theory and applications. *Neurocomputing*.

[15] Song Y., Zhang J. (2013). Automatic recognition of epileptic EEG patterns via Extreme Learning Machine and multiresolution feature extraction. *Expert Systems with Applications*.

[16] Yuan Q., Zhou W., Li S., Cai D. (2011). Epileptic EEG classification based on extreme learning machine and nonlinear features. *Epilepsy Research*.

[17] Qureshi M. N. I., Min B., Jo H. J., Lee B. (2016). Multiclass classification for the differential diagnosis on the ADHD subtypes using recursive feature elimination and hierarchical extreme learning machine: structural MRI study. *PLoS ONE*.

[18] Gao L., Cheng W., Zhang J., Wang J. (2016). EEG classification for motor imagery and resting state in BCI applications using multi-class Adaboost extreme learning machine. *Review of Scientific Instruments*.

[19] Tibshirani R. (2007). Regression shrinkage and selection via the Lasso Robert Tibshirani. *Journal of the Royal Statistical Society: Series B (Statistical Methodology)*.

[20] Friedman J., Hastie T., Tibshirani R. (2010). Regularization paths for generalized linear models via coordinate descent. *Journal of Statistical Software*.

[21] Huang G.-B., Zhou H., Ding X., Zhang R. (2012). Extreme learning machine for regression and multiclass classification. *IEEE Transactions on Systems, Man, and Cybernetics Part B: Cybernetics*.

[22] Cao J., Lin Z., Huang G.-B. (2010). Composite function wavelet neural networks with extreme learning machine. *Neurocomputing*.

[23] Huang G.-B., Ding X., Zhou H. (2010). Optimization method based extreme learning machine for classification. *Neurocomputing*.

[24] Bartlett P. L. (1998). The sample complexity of pattern classification with neural networks: the size of the weights is more important than the size of the network. *Institute of Electrical and Electronics Engineers. Transactions on Information Theory*.

[25] Shi L.-C., Lu B.-L. (2013). EEG-based vigilance estimation using extreme learning machines. *Neurocomputing*.

[26] Peng Y., Lu B.-L. (2014). Discriminative manifold extreme learning machine and applications to image and EEG signal classification. *Neurocomputing*.

[27] Liang N.-Y., Saratchandran P., Huang G.-B., Sundararajan N. (2006). Classification of mental tasks from EEG signals using extreme learning machine. *International Journal of Neural Systems*.

[28] Kim J., Shin H. S., Shin K., Lee M. (2009). Robust algorithm for arrhythmia classification in ECG using extreme learning machine. *Biomedical Engineering OnLine*.

[29] Karpagachelvi S., Arthanari M., Sivakumar M. (2012). Classification of electrocardiogram signals with support vector machines and extreme learning machine. *Neural Computing and Applications*.

[30] Huang W., Tan Z. M., Lin Z. A semi-automatic approach to the segmentation of liver parenchyma from 3D CT images with Extreme Learning Machine.

[31] Barea R., Boquete L., Ortega S., López E., Rodríguez-Ascariz J. M. (2012). EOG-based eye movements codification for human computer interaction. *Expert Systems with Applications*.

[32] Huang G., Huang G.-B., Song S., You K. (2015). Trends in extreme learning machines: a review. *Neural Networks*.

[33] Idrees B. M., Farooq O. Vowel classification using wavelet decomposition during speech imagery.

[34] Riaz A., Akhtar S., Iftikhar S., Khan A. A., Salman A. Inter comparison of classification techniques for vowel speech imagery using EEG sensors.

[35] Martin S., Brunner P., Iturrate I. (2016). Word pair classification during imagined speech using direct brain recordings. *Scientific Reports*.

[36] Pei X., Hill J., Schalk G. (2012). Silent communication: toward using brain signals. *IEEE Pulse*.

[37] Giraud A.-L., Poeppel D. (2012). Cortical oscillations and speech processing: emerging computational principles and operations. *Nature Neuroscience*.

[38] Towle V. L., Yoon H.-A., Castelle M. (2008). ECoG *γ* activity during a language task: differentiating expressive and receptive speech areas. *Brain*.

[39] Martin S., Brunner P., Holdgraf C. (2014). Decoding spectrotemporal features of overt and covert speech from the human cortex. *Frontiers in Neuroengineering*.

[40] Brumberg J. S., Nieto-Castanon A., Kennedy P. R., Guenther F. H. (2010). Brain-computer interfaces for speech communication. *Speech Communication*.

[41] Farwell L. A., Donchin E. (1988). Talking off the top of your head: toward a mental prosthesis utilizing event-related brain potentials. *Electroencephalography and Clinical Neurophysiology*.

[42] Krusienski D. J., Sellers E. W., Cabestaing F. (2006). A comparison of classification techniques for the P300 Speller. *Journal of Neural Engineering*.

[43] Yin E., Zhou Z., Jiang J., Chen F., Liu Y., Hu D. (2013). A novel hybrid BCI speller based on the incorporation of SSVEP into the P300 paradigm. *Journal of Neural Engineering*.

[44] Herff C., Schultz T. (2016). Automatic speech recognition from neural signals: a focused review. *Frontiers in Neuroscience*.

[45] Chakrabarti S., Sandberg H. M., Brumberg J. S., Krusienski D. J. (2015). Progress in speech decoding from the electrocorticogram. *Biomedical Engineering Letters*.

[46] Mohanchandra K., Saha S. (2016). A communication paradigm using subvocalized speech: translating brain signals into speech. *Augmented Human Research*.

[47] Ossmy O., Fried I., Mukamel R. (2015). Decoding speech perception from single cell activity in humans. *NeuroImage*.

